# Impacts of Circular RNAs on the Osteogenic Differentiation of Dental Stem Cells

**DOI:** 10.1155/sci/8338337

**Published:** 2025-05-08

**Authors:** Yang Wang, Meijie Tu, Huihui Gao, Shuli Deng

**Affiliations:** Stomatology Hospital, School of Stomatology, Zhejiang University School of Medicine, Zhejiang Provincial Clinical Research Center for Oral Diseases, Key Laboratory of Oral Biomedical Research of Zhejiang Province, Cancer Center of Zhejiang University, Engineering Research Center of Oral Biomaterials and Devices of Zhejiang Province, Hangzhou, China

**Keywords:** circular RNAs, dental stem cells, osteogenic differentiation, tissue engineering

## Abstract

Dental stem cells are widely viewed as good options for bone regeneration because of their ease of acquisition, innate ability to renew themselves, and ability to differentiate into different types of cells. However, the process of osteogenic differentiation of dental stem cells is orchestrated by an intricate system of regulatory mechanisms. Recent studies have demonstrated the critical impacts of circular RNAs (circRNAs) on osteogenic differentiation of dental stem cells. Exploring the roles and regulatory pathways of circRNAs in dental stem cells could identify novel targets and approaches for utilizing dental stem cell therapy in clinical settings. This review provides a comprehensive overview of the functions and mechanisms of circRNAs, with a particular focus on their expression patterns and regulatory roles in osteogenic differentiation of various dental stem cell types. Furthermore, this review discusses current research challenges in this field and proposes future directions for advancing our understanding of circRNA-mediated regulation in dental stem cell biology.

## 1. Introduction

**Figure 1:**
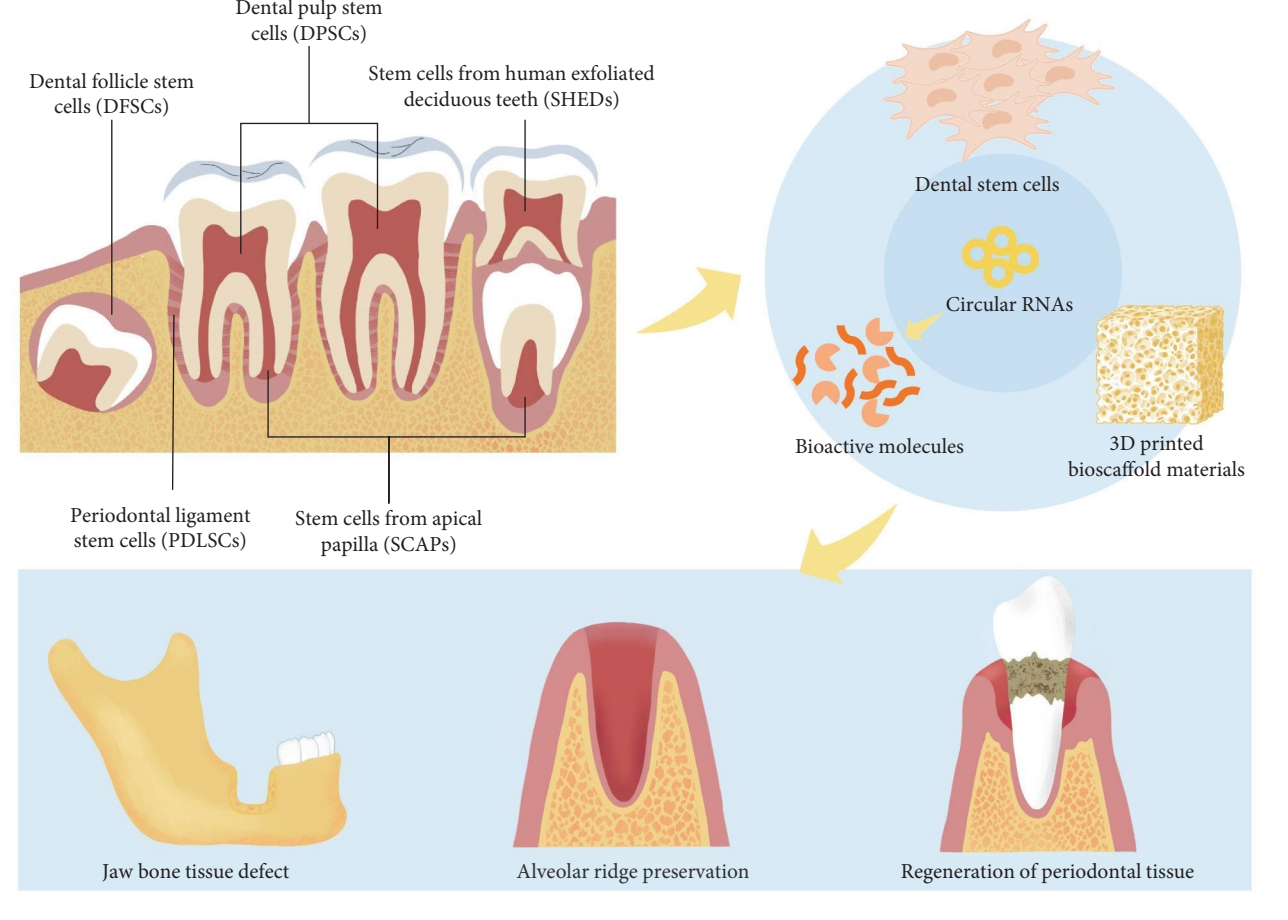
Application of dental stem cells for repairing maxillofacial bone defects.

Maxillofacial region tumors, injury, and inflammation frequently lead to bone tissue defects that strongly affect the ability of patients to chew and their appearance [1]. The economic burden of treating these defects is substantial, with current therapies often requiring invasive procedures and yielding suboptimal results [2]. Advances in tissue engineering have led to the utilization of stem cells, scaffold materials, and bioactive molecules to repair maxillofacial bone defects [3, 4]. With respect to tissue engineering, ideal stem cells should have robust proliferation and multidirectional differentiation capabilities and should be easily accessible for collection. While bone marrow mesenchymal stem cells (BMSCs) were the initial type to be discovered, their utilization in clinical applications is limited because of their invasive harvesting methods, low yields, and risk of infection at the donor site [5]. Instead, dental stem cells are highly desirable for tissue engineering applications because of their ease of access, high initial cell production, short doubling time, and impressive flexibility [6, 7]. These mesenchymal stem cells can also both renew themselves and differentiate into other types of cells, which makes them viable choices for repairing bone defects in the maxillofacial region [8]. Research has shown that dental stem cells offer advantages over BMSCs in terms of their differentiation capacity, immunogenicity, and immunosuppressive effects, making these cells more suitable for allogeneic stem cell therapy [9]. Various dental stem cell types (Figure 1), such as dental pulp stem cells (DPSCs) [10], stem cells from human exfoliated deciduous teeth (SHEDs) [11], periodontal ligament stem cells (PDLSCs) [12], stem cells from the apical papilla (SCAPs) [13], and dental follicle stem cells (DFSCs) [14], have been discovered. The process by which dental stem cells effectively differentiate into osteogenic cells is regulated by several physical, chemical, and biological variables. These different factors may stimulate specific signaling pathways and transcription factors. Nonetheless, the precise regulatory mechanisms remain unclear. Hence, a thorough investigation into the variables that direct and control the osteogenic differentiation capacity of dental stem cells is urgently needed.

Recent studies have highlighted the critical roles of circular RNAs (circRNAs) in regulating gene expression and cellular processes, including stem cell differentiation. CircRNAs constitute a novel category of noncoding RNAs and are distinguished by their closed-loop structures, which were initially observed in RNA viruses [15]. Advances in high-throughput sequencing and bioinformatics have revealed that unconventional RNA splicing is common for gene expression in eukaryotes [16]. Notably, circRNAs exhibit tissue- and cell-specific expression profiles in eukaryotes. Research has demonstrated their diverse regulatory functions in gene expression, implicating circRNAs in various physiological processes and human diseases, such as malignant tumors [17], cardiovascular diseases [18], and neurological disorders [19]. Furthermore, studies have shown the differential expression of circRNAs during dental stem cell differentiation and renewal, suggesting their potential to modulate stem cell behavior [20, 21]. Given the importance of dental stem cells in regenerative medicine, understanding how circRNAs influence osteogenic differentiation and tissue regeneration is highly important. This review provides a thorough examination of the impacts of circRNAs on osteogenic differentiation of different dental stem cells and their related mechanisms, illustrating the promise of circRNAs as a basis for stem cell therapy in regenerative medicine. Furthermore, we discuss the challenges and future directions in circRNA research, emphasizing the potential of these molecules in advancing regenerative medicine.

## 2. Characterization and Roles of circRNAs

**Figure 2:**
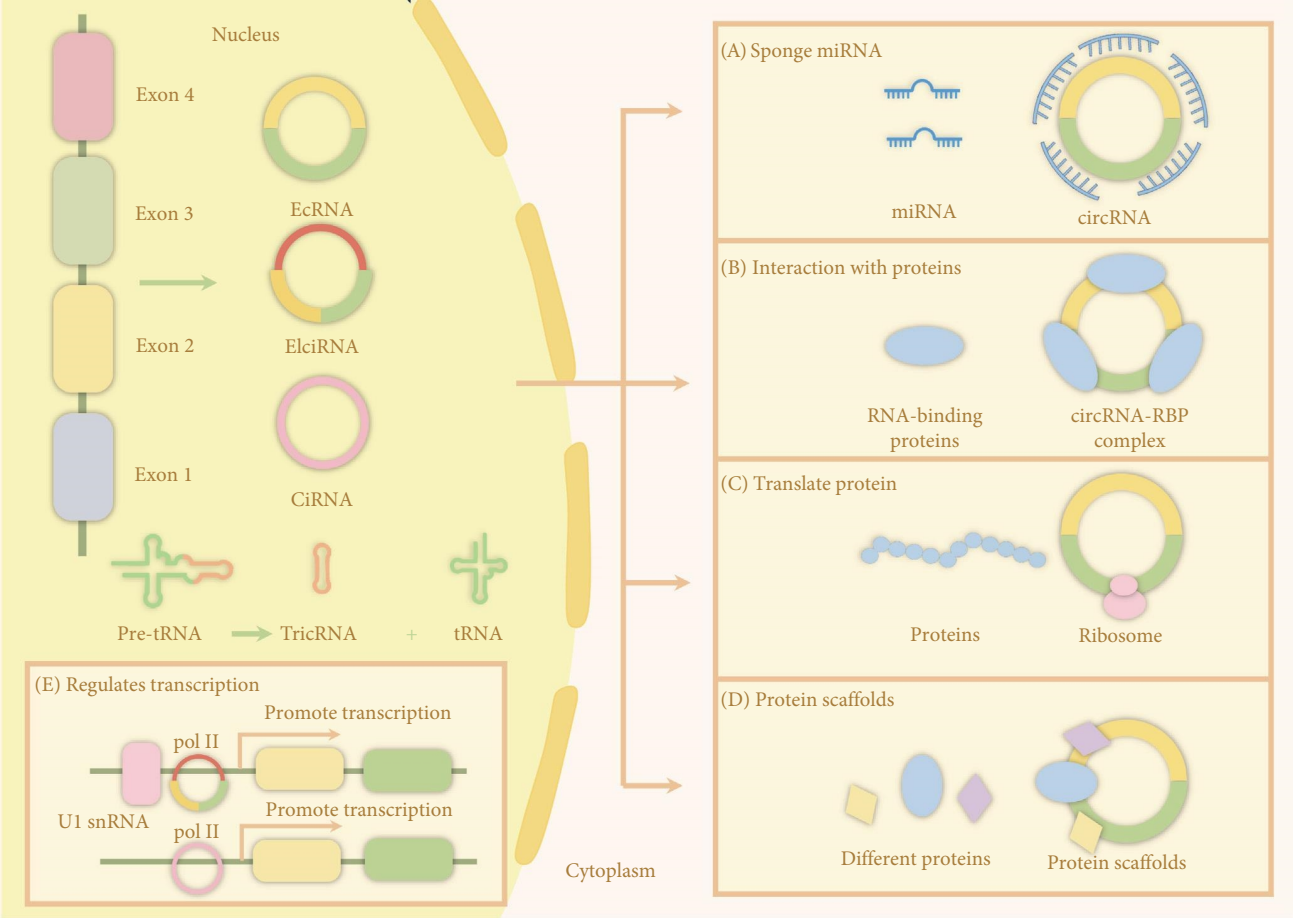
Biosynthesis and function of circRNAs.

circRNAs are unique covalent closed-loop structures that are formed by precursor mRNAs through reverse splicing between the 5′ and 3′ splice sites [22]. The circRNAs depicted in Figure 2 fall into four distinct types on the basis of their composition: exonic circRNAs, intronic circRNAs, exon‒intronic circRNAs, and tRNA‒intronic circRNAs, of which exon‒intronic circRNAs account for the majority [18]. The process of circRNA biosynthesis remains incompletely understood. However, several potential mechanisms have been proposed, including exon jumping or lasso-driven cyclization, intron pairing-driven cyclization, and RNA-binding protein (RBP)-related pairing-driven cyclization [23–25]. Notably, circRNA biogenesis competes with the splicing of linear precursor mRNAs, and this competition is regulated by various factors, including transcription factors, cis-acting elements, trans-acting factors, and RBPs [18]. After being biosynthesized, circRNAs, depending on their length, undergo translocation to the cytoplasm, which is mediated by the spliceosomal RNA deconvertase DDX39B and the ATP-dependent RNA deconvertase DDX39A [26]. An additional nuclear export pathway that involves the IGF2BP1, exportin-2, and Ran-GTP proteins has also been identified [27]. Research has shown that circRNA export increases when the nuclear Ran-GTP gradient increases and is hindered when the gradient decreases. Furthermore, the association of exportin-2 and IGF2BP1 with circRNAs, which is facilitated by Ran-GTP, results in the formation of a complex that aids in the transport of circRNAs to the cytoplasm. Once in the cytoplasm, circRNAs are protected from degradation by RNase R enzymes and other nucleic acid exonucleases because of their unique circular structure, which maintains their stability [28]. Nevertheless, the processes underlying the degradation of circRNAs remain poorly understood, with only a limited number of recent studies proposing potential hypotheses for further investigation. One such mechanism involves circRNAs with m6A modifications, which render them susceptible to degradation by specific nucleic acid endonucleases [29]. These enzymes cleave circRNAs, leading to their breakdown and removal from the cellular environment. Another pathway involves argonaute-mediated cleavage, a protein-dependent process that facilitates circRNA degradation and prevents excessive accumulation [30]. Moreover, cytokinesis, the division of the cytoplasm during cell division, may serve as a mechanism for circRNA clearance, contributing to the maintenance of cellular homeostasis and proper gene expression regulation [31].

circRNAs have various biological functions, among which the most widely studied is their ability to act as microRNA (miRNA) sponges [15]. As competing endogenous RNAs, circRNAs can bind to and inhibit the activity of miRNAs, reducing their effects on downstream target genes. One notable example is CDR1as, which contains numerous binding sites for miR-7 and was shown to affect gene expression in neural tissues [32–34]. This interaction indirectly affects zebrafish midbrain development and can affect disease processes [35, 36]. In addition to functioning as miRNA sponges, circRNAs act as protein sponges or scaffolds, facilitating protein interactions and modulating gene expression [37–39]. For example, circHIPK3 was shown to regulate cardiomyocyte senescence by promoting the degradation of specific proteins through interactions with E3 ubiquitin ligases [40]. Although their translation into proteins is generally limited, a small percentage of circRNAs can be translated to generate proteins or polypeptides by binding to ribosomes [41, 42]. In addition, intronic circRNAs and exon‒intronic circRNAs function as cis-acting as transcriptional regulators in the nucleus. For example, EIciEIF3J and EIciPAIP2 regulate RNA polymerase II activity and promote gene transcription by combining with the U1 small nuclear ribonucleoprotein [43]. Overall, circRNAs constitute a critical class of molecules that are involved in transcriptional control, with implications for various cellular processes, including stem cell function. The significance of these molecules in the regulation of dental stem cells also warrants further investigation.

## 3. Roles of circRNAs in the Osteogenic Differentiation of Dental Stem Cells

### 3.1. DPSCs

**Figure 3:**
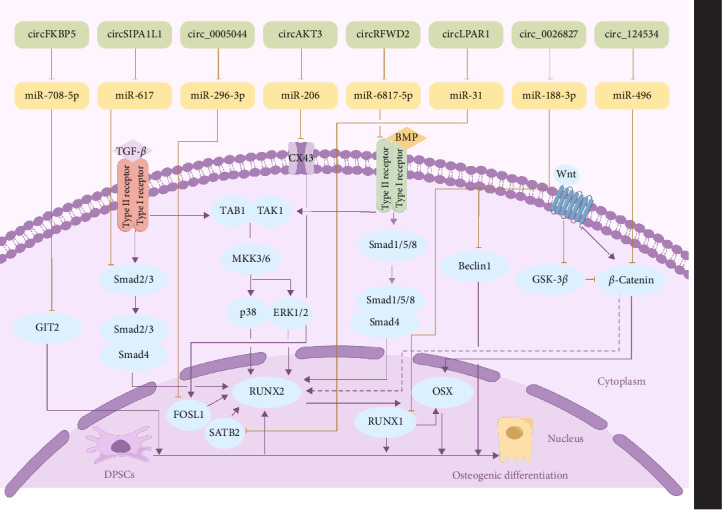
Mechanisms of circRNAs in regulating osteogenic differentiation of DPSCs.

DPSCs, which develop from dental papilla, can be obtained from adult dental pulp tissue via enzymatic treatment [44]. In cases of severe tooth decay or dental pulp exposure, DPSCs are essential for regulating the response of the body to inflammation and promoting the formation of reparative dentin [45]. DPSCs were the initial type of stem cells found in dental research. These cells demonstrate a strong capacity for self-renewal and the potential to differentiate in multiple directions [5]. Therefore, these cells are often used as sources of mesenchymal stem cells for bone tissue engineering. During the osteogenic differentiation of DPSCs, a total of 86 were found to be differentially expressed, 57 of which presented increased expression. Further research demonstrated that upregulated circAKT3 indirectly increased CX43 expression by binding to miR-206 to promote the osteogenic differentiation of DPSCs [46]. Another study revealed that circ_0026827 interacted with miR-188-3p and modified the Beclin1 and RUNX1 signaling pathways to promote the osteoblast differentiation of DPSCs [47]. Furthermore, both circAKT3 and circ_0026827 promoted ectopic bone formation in vivo, which highlights their potential in bone regenerative therapy. CircRNAs are predominantly found in the cytoplasm, but they can also be secreted via exosomes, affecting the function and state of nearby cells [31]. In a study by Xie et al., osteogenic-induced DPSC-derived exosomes were shown to promote the osteogenic differentiation of recipient DPSCs. These exosomes contained circLPAR1, which binds to miR-31, suppressing its ability to downregulate osteogenic differentiation-related genes, such as SATB2 and RUNX2 [48]. Exosomal circRNAs create a reinforcing loop, which increases their ability to induce themselves and neighboring cells to promote successful osteogenic differentiation. Notably, the functionality of stem cells is compromised in an inflammatory setting [49, 50]. Liang et al. demonstrated that the upregulation of circFKBP5 mitigated lipopolysaccharide (LPS)-induced apoptosis, inflammation, proliferation inhibition, and suppression of osteogenic differentiation of DPSCs. These protective mechanisms are linked to the miR-708-5p/GIT2 axis, suggesting potential therapeutic strategies for treating inflammation-related bone diseases with circRNAs [51] (Figure 3, Table 1).

### 3.2. SHEDs

With the discovery of DPSCs, Miura et al. succeeded in isolating SHEDs from the pulp tissue of deciduous teeth [11, 57]. There were no notable differences in the cellular or biological characteristics of SHEDs when the resorption of deciduous tooth roots was less than 2/3 of the total root length. However, if root resorption exceeded 2/3 of the length, the success rate of SHED isolation significantly decreased because of the limited availability of pulp tissue in deciduous teeth, leading to a reduction in the osteogenic differentiation capacity [58, 59]. The abundance, accessibility, minimal invasiveness, and limited ethical restrictions on the use of SHEDs make them highly suitable for application in the field of regenerative medicine. Empirical evidence has indicated that SHEDs have a greater capacity for proliferation or survival than other types of dental stem cells do [60]. Moreover, SHEDs possess substantial immunomodulatory and immunosuppressive capabilities [60, 61]. Although research on the regulatory mechanisms of miRNAs and long noncoding RNAs (lncRNAs) in the osteogenic differentiation of SHEDs is limited, recent studies have provided important insights [62–64]. For example, miR-27b-5p hinders the osteogenic differentiation of SHEDs by targeting BMPR1A and inhibiting BMP signaling [65]. Thus, miRNAs, lncRNAs, and circRNAs work together in a complex network to regulate the osteogenic differentiation of SHEDs. A thorough exploration of these regulatory mechanisms may promote our understanding of bone development and diseases, offering innovative approaches to bone regeneration. This research shows promise for advancing regenerative medicine and improving outcomes for patients in need of bone regeneration therapy.

### 3.3. PDLSCs

PDLSCs, which are derived from the periodontal ligament, have shown promising capabilities in regenerating periodontal tissues, including periodontal ligament–cementum–like structures, blood vessels, peripheral nerves, and alveolar bone [66]. These cells are highly regarded for their capacity to regenerate themselves and differentiate in multiple directions, along with being easily accessible and having low in immunogenicity [67–69]. Research findings have demonstrated that the levels of circRNAs in PDLSCs are altered during osteogenic differentiation and biomineralization. Through the construction of a circRNA–miRNA–mRNA network, circRNAs were found to have a strong effect on the regulation of osteogenic differentiation of PDLSCs [70, 71] (Table 2). Specifically, the circRNA CDR1as was found to block miR-7, thereby promoting osteogenic differentiation through the activation of the pSMAD1/5/8 and p38 MAPK pathways [72]. Additionally, CDR1as can directly interact with miR-7 and upregulate KLF4 expression in PDLSCs to maintain their pluripotency [74]. Ye et al. [75] provided evidence that circFAT1 affected osteogenic differentiation of PDLSCs by functioning as a miRNA sponge for miR-4781-3p, which specifically targets SMAD5. Furthermore, the study revealed that circ-SPATA13 promoted osteogenic differentiation of PDLSCs by acting as a molecular sponge for miR-485-5p_R + 1, which in turn upregulated BMP7 expression [78]. In vivo experiments confirmed that overexpression of circ-SPATA13 enhances bone formation in a rat skull defect model. These results substantially advance our knowledge of the mechanism underlying osteogenic differentiation of PDLSCs and could lead to innovative therapeutic approaches in the future.

Inflammation or a hypoxic microenvironment in local tissues can hinder the regenerative abilities of stem cells [96]. Several studies have highlighted the roles of circRNAs in exacerbating inflammation, oxidative stress, and apoptosis in PDLSCs, thereby promoting the progression of periodontitis. For example, the levels of circBIRC6 were found to increase in PDLSCs and periodontitis-affected tissues upon exposure to inflammatory substances. However, circBIRC6 silencing was shown to increase the bone-forming ability of PDLSCs under inflammatory conditions. Subsequent investigations confirmed that circBIRC6 suppressed the osteogenic capacity of PDLSCs by increasing PTEN levels through interaction with miR-543 in inflammatory environments, which impeded osteogenesis via negative regulation of the PI3K/AKT/mTOR axis [76]. Similarly, under hypoxic conditions, circCDK8 obstructed the bone-forming process of PDLSCs by increasing autophagy through the mTOR pathway [77]. Additionally, circPVT1 was significantly upregulated in periodontitis tissues and LPS-treated PDLSCs, in which it promoted inflammation and oxidative stress by sponging miR-24-3p and upregulating HIF1AN, leading to the inhibition of the NRF-2/HO-1 pathway [80]. Furthermore, a certain circRNA can affect the function of PDLSCs through multiple mechanisms. Studies have shown that circ_0099630 is upregulated in periodontitis and inhibits periodontal PDLSC proliferation and osteogenic differentiation while promoting apoptosis. This circRNA sponges miR-409-3p and miR-940 and upregulates TLR4 and TRAF6, which activate the NF-*κ*B pathway and exacerbate inflammation and PDLSC damage [83, 84]. Knockdown of circ_0099630 restores PDLSC function, reduces inflammation, and enhances osteogenic differentiation, making it a potential therapeutic target for periodontitis. Circ_0084054 was identified as a key player in diabetic periodontitis; it aggravated inflammation and oxidative stress in PDLSCs by regulating the miR-508-3p/PTEN axis, leading to the inhibition of AKT phosphorylation and an increase in apoptosis [86]. Moreover, circFOXO3 was found to be implicated in periodontitis by downregulating ITG*β*6 expression through the miR-141-3p/FOXO3/JunB signaling pathway, which further impaired PDLSC function and contributed to periodontal tissue destruction [81]. Collectively, these findings suggest that circRNAs can significantly impair PDLSC function by modulating various miRNA/mRNA axes, thereby contributing to chronic inflammation and tissue destruction, which are characteristic features of periodontitis. Targeting these circRNA-mediated pathways may offer novel therapeutic strategies for managing periodontitis.

Thus, preserving the bone-forming potential of PDLSCs in environments affected by inflammation or low oxygen levels is crucial for the effective treatment of bone loss in patients with periodontitis. Research has shown that CDR1as can activate the ERK signaling pathway by sponging miR-7, thereby attenuating the inhibitory effect of LPS on the proliferation of PDLSCs [73]. Furthermore, different circRNAs can regulate the same gene through different miRNA-mediated pathways to control the function of PDLSCs. For example, studies have demonstrated that circ_0062491 and circ_0085289 are downregulated in periodontitis and LPS-induced PDLSCs. Overexpression of circ_0062491 alleviates LPS-induced apoptosis and inflammation in PDLSCs by sponging miR-498, which directly targets SOCS6, whereas circ_0085289 exerts similar protective effects by absorbing let-7f-5p, another miRNA that negatively regulates SOCS6 [91, 95]. These findings illustrate that two different circRNAs, circ_0062491 and circ_0085289, can converge on the same gene, SOCS6, through distinct miRNA interactions, underscoring the multifaceted regulatory roles of circRNAs in periodontitis and their potential as therapeutic targets. Consequently, exploring circRNAs as potential therapeutic targets for regulating the osteogenic differentiation of PDLSCs could be essential for promoting periodontal regeneration.

### 3.4. SCAPs

SCAPs, which are present in the dental papilla at the root apex of young permanent teeth, are crucial for root development [97]. These stem cells possess mesenchymal stem cell markers and have the capacity for proliferation, self-renewal, and multidirectional differentiation [98]. Research has indicated that circRNA expression in SCAPs changes significantly during osteogenic differentiation. This differential gene expression is linked to various signaling pathways that are related to osteogenesis and stem cell pluripotency, emphasizing the regulatory function of circRNAs in the differentiation process [99, 100]. For example, circSIPA1L1 has been identified as a regulator of ALPL expression, promoting the osteogenic differentiation of SCAPs by binding to miR-204-5p [101]. ALPL, known for its role in bone and tooth development, is crucial for maintaining normal bone health [102]. CircSIPA1L1 was found to increase ALP expression, which can offer new perspectives on bone regeneration and potential treatments for hypophosphatasia. Furthermore, circ-ZNF236 can upregulate LGR4 expression to increase autophagy and promote osteogenic differentiation of SCAPs [103]. These results indicate the possibility of using circRNAs as therapeutic targets to increase osteogenic differentiation of SCAPs.

### 3.5. DFSCs

DFSCs are situated in the dental follicle tissue of the tooth germ and are derived from the neural crest [104]. These cells are considered direct precursor cells of periodontal tissues that have the ability to generate the cementum, periodontal ligament, and alveolar bone in advanced tooth development stages [105]. Examination of the expression patterns of circRNAs throughout the process of osteogenic differentiation of rat DFSCs demonstrated circRNA involvement in regulating essential signaling pathways, such as the MAPK and TGF-*β* pathways. This research further confirmed that circFgfr2 promoted osteogenesis by acting as a miR-133 sponge to upregulate BMP6 expression [106]. Despite these findings, the knowledge on the functions of circRNAs in the osteogenic differentiation of DFSCs is limited. These findings emphasize the need for future exploration to uncover the intricate functions played by circRNAs in this process, thereby offering novel perspectives for tissue regeneration.

## 4. Summary and Prospects

The utilization of dental stem cells in the fields of tissue engineering and bone regeneration shows promise. The process of osteogenic differentiation of dental stem cells is complex and requires a thorough understanding of its regulatory mechanisms. Recent research has highlighted the critical functions of circRNAs in this process. This review explores the functions of circRNAs in the process of osteogenic differentiation of dental stem cells. While sequencing approaches have revealed the presence of many differentially expressed circRNAs during osteogenesis of dental stem cells, our understanding of their functions and regulatory mechanisms is still in its early stages. Current studies focus on how circRNAs regulate gene expression in odontogenic stem cells through binding to specific miRNAs. However, data concerning other mechanisms by which circRNAs may regulate osteogenesis are lacking. New technologies, such as single-cell sequencing and digital spatial analysis, may help further identify and characterize circRNAs. Given that circRNAs are involved in various biological processes and diseases, these molecules are of interest for potential gene therapy applications. Future studies are needed to examine the involvement of circRNAs in dental stem cell osteogenesis and their potential use in bone regeneration. A more thorough understanding of circRNA functions and regulatory mechanisms is necessary to elucidate their therapeutic potential in bone tissue engineering.

Despite the promising potential of circRNAs, their clinical application faces several significant challenges. Efficient production and purification of circRNAs remain critical, as current methods often leave residual fragments or require extensive steps to remove impurities, which hinders scalability. Another major hurdle is the effective delivery of circRNAs to target tissues. Current delivery systems, such as lipid nanoparticles (LNPs), exosomes, and virus-like particles (VLPs), help protect circRNAs and enable their targeted delivery; however, each system has its own limitations. LNPs are efficient but primarily target the liver and have poor endosomal escape capability; exosomes are biocompatible but difficult to produce at scale; and VLPs are highly immunogenic and sensitive to environmental conditions. Future research should focus on developing innovative delivery systems that overcome these limitations. Although circRNAs are less immunogenic than linear mRNAs, they can still trigger innate immune responses, potentially compromising their efficacy. Strategies to further reduce immunogenicity, such as RNA modifications, need exploration. Addressing these challenges—production, delivery, immunogenicity, and expression control—is vital to fully harness the therapeutic potential of circRNAs. Continued innovation in these areas will pave the way for their successful clinical translation.

## Figures and Tables

**Table 1:** The regulatory effects and mechanisms of circRNAs on osteogenic differentiation of dental pulp stem cells.

circRNAs	Sponge	Target gene	Function	References
circ_124534	miR-496	*β*-catenin	Promote osteogenic differentiation	[52]
circ_0026827	miR-188-3p	Beclin1 and RUNX1	Promote osteogenic differentiation	[47]
circSIPA1L1	miR-617	SMAD3	Promote osteogenic differentiation	[53]
circLPAR1	miR-31	SATB2	Promote osteogenic differentiation	[48]
circAKT3	miR-206	CX43	Promote osteogenic differentiation	[46]
circRFWD2	miR-6817-5p	BMPR2	Promote osteogenic differentiation	[54]
circFKBP5	miR-708-5p	GIT2	Protect against LPS-induced apoptosis, inflammation, and osteogenic differentiation inhibition	[51]
circ_0005044	miR-296-3p	FOSL1	Promote osteogenic differentiation	[55]
circ_0036872	miR-143-3p	IGF2	Promote osteogenic differentiation	[56]

**Table 2:** The regulatory effects and mechanisms of circRNAs on the function of periodontal ligament stem cells.

circRNAs	Sponge	Target gene	Function	References
CDR1as	miR-7	GDF5	Promote osteogenic differentiation	[72]
CDR1as	miR-7	ERK	Promote cell proliferation	[73]
CDR1as	miR-7	KLF4	Maintain stemness	[74]
circFAT1	miR-4781-3p	SMAD5	Promote osteogenic differentiation	[75]
circBIRC6	miR-543	PTEN	Inhibit osteogenic differentiation	[76]
circCDK8	—	mTOR	Inhibit osteogenic differentiation and promote autophagy	[77]
circ-SPATA13	miR-485-5p_R + 1	BMP7	Promote osteogenic differentiation	[78]
circ_0003764	—	—	Inhibit osteogenic differentiation and proliferation	[79]
circPVT1	miR-24-3p	HIF1AN	Promote apoptosis, inflammation, and oxidative stress	[80]
circFOXO3	miR-141-3p	FOXO3	Promote inflammation	[81]
circ_0087199	miR-527	TLR4	Promote apoptosis, inflammation, and oxidative stress	[82]
circ_0099630	miR-409-3	TLR4	Inhibit proliferation, promote cell apoptosis, and inflammation	[83]
circ_0099630	miR-940	TRAF6	Promote apoptosis and inflammation	[84]
circ_0099630	miR-212-5p	SPRY1	Inhibit proliferation and osteogenic differentiation	[85]
circ_0084054	miR-508-3p	PTEN	Promote inflammation and oxidative stress	[86]
circRASA2	miR-543	TRAF6	Inhibit proliferation, migration, and osteogenic differentiation	[87]
circ_0097010	miR-769-5p	KLF6	Promote apoptosis and inflammation	[88]
circ_0138960	miR-518a-5p	HDAC6	Promote apoptosis and inflammation	[89]
circ_0138959	miR-495-3p	TRAF6	Inhibit proliferation and osteogenic differentiation, promote apoptosis, and inflammation	[90]
circ_0062491	miR-498	SOCS6	Inhibit apoptosis and inflammation	[91]
circ_0003948	miR-144-3p	NR2F2	Promote proliferation and inhibit apoptosis	[92]
circ_0066881	miR-144-5p	RORA	Inhibit apoptosis and inflammation	[93]
circ_0081572	miR-378h	RORA	Inhibit apoptosis and inflammation	[94]
circ_0085289	let-7f-5p	SOCS6	Inhibit apoptosis and inflammation	[95]

## Data Availability

The data sharing is not applicable to this article as no datasets were generated or analyzed during the current study.
